# Serum Semaphorin Alterations in Psoriasis: Links to Metabolic Status Rather than Disease Severity

**DOI:** 10.3390/metabo16030190

**Published:** 2026-03-12

**Authors:** Anna Baran, Anna Stepaniuk, Justyna Magdalena Hermanowicz, Beata Sieklucka, Krystyna Pawlak, Dariusz Pawlak, Iwona Flisiak

**Affiliations:** 1 Department of Dermatology and Venereology, Medical University of Bialystok, Zurawia 14 St., 15-540 Bialystok, Poland; anna.baran@umb.edu.pl (A.B.); iwona.flisiak@umb.edu.pl (I.F.); 2 Department of Pharmacodynamics, Medical University of Bialystok, Mickiewicza 2C St., 15-540 Bialystok, Poland; justyna.hermanowicz@umb.edu.pl (J.M.H.); dariusz.pawlak@umb.edu.pl (D.P.); 3 Department of Monitored Pharmacotherapy, Medical University of Bialystok, Mickiewicza 2C St., 15-222 Bialystok, Poland; beata.sieklucka@umb.edu.pl (B.S.); krystyna.pawlak@umb.edu.pl (K.P.)

**Keywords:** semaphorins, Sema3A, Sema3E, Sema4A, Sema4D, Sema7A, atherosclerosis, psoriasis, comorbidities, metabolic syndrome, systemic therapy

## Abstract

Introduction: Psoriasis is an autoimmune systemic disease of not entirely understood pathogenesis. It remains a significant therapeutic challenge and, due to its various comorbidities, has a remarkable detrimental effect on patients’ wellbeing. Semaphorins (Sema) are a group of transmembrane, cell surface-attached and secretory proteins that might play an important role in psoriasis due to their presence on keratinocytes and the ability to stimulate the proinflammatory cytokine production. Aims: The study aimed to assess the concentration of Sema3A, Sema3E, Sema4A, Sema4D and Sema7A in serum samples of psoriatic patients and explore the correlation with disease activity and clinical and metabolic status. Materials and Methods: The study involved 60 patients with plaque psoriasis and 30 healthy volunteers matched for gender, age, and BMI. Results: The mean serum Sema3A, Sema3E and Sema4D levels were significantly higher in patients with psoriasis than controls (*p* < 0.01, *p* < 0.05 and *p* < 0.05, respectively). Contrarily, Sema4A and Sema7A were significantly lower (*p* < 0.001 and *p* < 0.05 respectively). Significant positive correlation between Sema3A and UREA was noted. Sema3A levels were significantly higher in moderately ill and overweight patients (*p* < 0.05, *p* < 0.01, respectively) and in patients with longer-lasting psoriasis and male patients compared to controls (both *p* < 0.05). Sema3E significantly negatively correlated with HDL and glucose levels. Sema4A was significantly lower in moderately and severe psoriatic patients (*p* < 0.0001, *p* < 0.01, respectively). Sema7A was significantly higher in moderately ill and overweight patients (*p* < 0.05, *p* < 0.01, respectively) and significantly lower in male patients and in those with longer lasting disease than in controls. None of the semaphorins correlated with psoriasis severity, total BMI, psoriasis duration and age. Conclusions: Psoriatic patients exhibited distinct alterations in circulating semaphorins, with significantly increased serum Sema3A, Sema3E and Sema4D, and reduced Sema4A and Sema7A compared with healthy subjects. Selected semaphorins demonstrated associations with metabolic parameters and patient characteristics, although none can serve as marker of disease severity. The findings indicate that semaphorins may reflect psoriasis-related systemic disturbances, but further studies are required to explore their potential with disease-associated metabolic or clinical profiles.

## 1. Introduction

Psoriasis is a common inflammatory disease that affects 2 to 3% of the worldwide population [1]. The exact pathogenesis remains unclear but genetic factors and genes such as HLA-C*06:02 and environmental stimuli including stress, alcohol consumption, trauma play roles in its development [2]. Psoriasis is linked to various comorbidities such as atherosclerosis, hypertension, type 2 diabetes mellitus (DM), obesity, non-alcoholic fatty liver disease (NAFLD), chronic kidney disease (CKD), cardiovascular diseases (CVDs), depression and anxiety, which significantly decrease the patients’ quality of life and lead to additional costs for healthcare systems worldwide [3,4].

Semaphorins are a group of proteins that include transmembrane, cell surface-attached and secretory molecules and are responsible for morphological differentiation and the movement of various cell types, such as immune, endocrine, reproductive or renal [5,6]. To date, over 30 semaphorins have been identified and grouped into eight different classes according to the structure and presence among different phylia [5,6]. It has been noted that clinically, class 3 plays particularly significant role and especially semaphoring 3A, which is believed to lower IL-4 and IL-10 production, epidermal thickness and cutaneous blood vessels, has been identified as an important molecule in disorders including malignancies, autoimmune conditions and allergies [5,7]. Furthermore, it positively correlates with IL-17 and participates in the differentiation of Th-17 cells [8,9]. Another semaphorin from this class, Sema3E, has been linked to CVDs, asthma and malignant processes including breast and colon cancers or melanoma [10]. Class 4 includes membrane semaphorins such as Sema4A and Sema4D, which have been linked to autoimmune conditions such as multiple sclerosis [11]. Furthermore, semaphorin 4D is known to stimulate angiogenesis and some data suggest it may play a role in malignant processes—such as tumor progression and metastases [12]. Semaphorin 7A, the only one classified in the seventh subfamily, is believed to promote the production of IL-8 and monocytic activation, which increases inflammation and has an impact on bone remodeling and atherosclerosis development by stimulating neovascularization, which in turn is a common comorbidity among patients with psoriasis [13,14].

Some semaphorins have been studied in psoriasis; however, the data are sparse [5]. Lower concentration of semaphorin 3A in the epidermis of patients with psoriasis has been demonstrated [5]. Moreover, it has been suggested that deviations in Sema3A expression in psoriatic skin can lead to pruritus often accompanying symptoms [5,15]. Sema7A, present on keratinocytes, is understood to participate in the inflammatory process in psoriasis by activating monocytes by keratinocytes [5,16]. Despite semaphorins’ important role in keratocytes’ activation and vascularization and their multidirectional influence, this group of molecules seems to be still under-researched in psoriasis and may contribute to a better understanding of the pathogenesis of comorbidities associated with this disease.

## 2. Materials and Methods

### 2.1. Study Design and Participants

This study was conducted at the Department of Dermatology, Medical University of Bialystok, and enrolled 60 adult patients (≥18 years) with clinically confirmed plaque psoriasis. A comparison group of 30 dermatologically healthy individuals was included and matched to the patient group by age, sex, and body mass index (BMI). Individuals were excluded if they were pregnant or breastfeeding; had followed special diets; received anti-psoriatic therapy within four weeks before inclusion; or had a history of hypertension, chronic renal or cardiac insufficiency, liver disorders, acute or chronic infections, autoimmune diseases other than psoriasis, or malignancy diagnosed either currently or within the previous five years.

Disease severity was evaluated at hospital admission using the Psoriasis Area and Severity Index (PASI). All assessments were performed by a single investigator to ensure consistency. Based on PASI scores, patients were classified into three categories: mild psoriasis (PASI < 10; *n* = 9), moderate psoriasis (PASI 10–20; *n* = 41), and severe psoriasis (PASI > 20; *n* = 10).

Body mass index was determined using the standard formula (kg/m^2^). Participants were additionally stratified according to disease duration (≤15 years vs. >15 years). For BMI-based analyses, subjects were allocated to the following groups: controls (group 0); normal-weight psoriasis patients (BMI 18.5–24.9; group 1); overweight psoriasis patients (BMI 25–29.9; group 2, *n* = 10); and obese psoriasis patients (BMI ≥ 30; group 3).

Blood samples were obtained from all participants, and serum was stored at −80 °C until biochemical evaluation. Laboratory analyses included high-sensitivity C-reactive protein (hs-CRP), complete blood count, fasting glucose, and lipid profile (total cholesterol, HDL, LDL, and triglycerides), as well as parameters reflecting renal and hepatic function.

The study protocol received approval from the Bioethics Committee of the Medical University of Bialystok (approval no. APK.002.358.2022) and adhered to the ethical standards of the Declaration of Helsinki. Written informed consent was obtained from all participants prior to participation in the study.

### 2.2. Serum Collection

Fasting blood samples were collected using vacuum tubes and subsequently centrifuged for 10 min at 2000× *g*. The resulting serum was aliquoted and stored at −80 °C until analysis. Routine laboratory methods were employed to determine standard biochemical indices. Serum levels of Sema3A, Sema3E, Sema4A, Sema4D, and Sema7A were determined using enzyme-linked immunosorbent assay (ELISA) kits from Cloud Clone^®^ (Houston, TX, USA; catalog numbers: SEL917Hu, SEL920Hu, SEL921Hu, SEB430Hu, and SEB448Hu, respectively). Only serum samples for Sema4D required a 500-fold dilution. In the remaining ELISA kits, serum samples were not diluted. The measurement principle was based on a sandwich reaction. The microplates provided in these kits were pre-coated with an antibody specific to the appropriate Sema. In the first stage, 100 µL of standards and samples were added to each well of a plate coated with antibodies specific to Sema3A, Sema3E, Sema4A, Sema4D, and Sema7A, respectively. Subsequently, the plate was incubated for 1 h at 37 °C. Then, 100 µL of Detection Reagent A was added to the plate and incubated for 60 min at 37 °C. In the next step, the plate was rinsed three times using an Elx-50 automated microplate washer (BioTEK^®^, Santa Clara, CA, USA). Next, 100 µL of Detection Reagent B was added to each well, and incubated for 30 min at 37 °C. After the incubation period, the plate was washed five times. In the next step, 90 µL of substrate solution was added to each well and incubated for 20 min at 37 °C. After the addition of the substrate solution, only those wells that contained the appropriate Sema, biotin-conjugated antibody, and enzyme-conjugated Avidin exhibited a change in color. Next, the enzyme–substrate reaction was terminated by the addition of 50 µL of STOP Solution (sulphuric acid solution). The Sema3A, Sema3E, Sema4A, Sema4D, and Sema7A concentrations were measured spectrophotometrically at a wavelength of 450 nm using a Multiscan FC microplate reader (ThermoScientific^®^, Waltham, MA, USA). The concentration of Semas in the samples was determined by comparing the O.D. of the samples to the standard curve created in the SkanIt software ver 2.x (ThermoScientific^®^, Waltham, MA, USA). The levels of Sema3A, Sema3E, Sema4A, and Sema7A were measured in ng/mL, whereas Sema4D was measured in pg/mL. The minimum detectable doses of Sema3A, Sema3E, Sema4A, and Sema7A were less than 0.62ng/mL, 0.117ng/mL, 0.113ng/mL, 12.1pg/mL, and 0.061ng/mL, respectively. The intra-assay coefficients of variation (CVs) for all Semas were CV < 10%, whereas the inter-assay coefficients of variation were CV < 12%. All laboratory measurements were conducted by a single trained operator under standardized laboratory conditions.

### 2.3. Statistical Analysis

The Shapiro–Wilk test was applied to evaluate the normality of data distribution. Variables exhibiting a normal distribution were analyzed using Student’s t-test or one-way analysis of variance (ANOVA) and are reported as mean ± SD. Non-normally distributed variables were summarized as a median (range) and examined using the non-parametric Mann–Whitney or Kruskal–Wallis tests. Associations between the studied variables were determined using Spearman’s rank correlation. Statistical analyses were conducted with GraphPad Prism version 9.4. A *p*-value < 0.05 was considered indicative of statistical significance.

## 3. Results

The baseline characteristics of the participants are presented in Table 1 and Table 2.

**Table 1:** Basic characteristics of the participants.

Parameter	Controls (*n* = 30)	Psoriasis (*n* = 60)
Sex (M/F)	20/10	39/21 NS
Age [years]	47 ± 2.5	49 ± 2.3 NS
Height [m]	1.7 ± 0.01	1.7 ± 0.01 NS
Weight [kg]	78 ± 3	84 ± 2 NS
BMI	25.7 ± 0.77	27.6 ± 0.8 NS

NS, non-significant.

**Table 2:** Correlation between laboratory parameters and patients with psoriasis.

Parameter	Psoriasis (*n* = 60)
GLU	96.86 mg/dL
CRP	7.55 mg/dL
RBC	4.59 mln
WBC	7.12 × 10^3^/μL
HDL	48.84 mg/dL
TG	156.56 mg/dL
LDL	101.2 mg/dL

### 3.1. Semaphorin 3A

**Figure 1:**
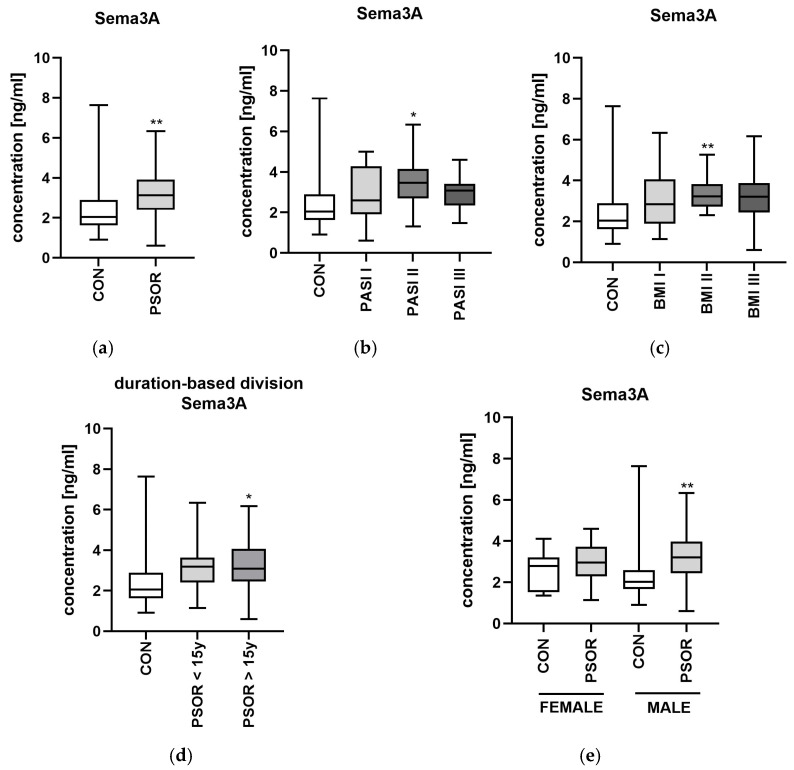
Serum semaphorin 3A concentration in patients in total (**a**) and divided regarding PASI (**b**), BMI (**c**), psoriasis duration (**d**), and gender (**e**) compared to controls. */** means a statistically significant difference compared to the control group with *p* < 0.05/0.001. CON *n* = 30; PSOR *n* = 60.

The serum concentration of semaphorin 3A was significantly higher in patients with psoriasis than controls (*p* < 0.01) (Figure 1a–d).

Sema3A levels were significantly higher in moderately ill and overweight patients (*p* < 0.05, *p* < 0.01, respectively) (Figure 1b,c). They were significantly higher in patients with longer-lasting psoriasis and male patients compared to controls (*p* < 0.05) (Figure 1d,e). However, no important differences between sex or disease duration within the study group were noted.

**Figure 2:**
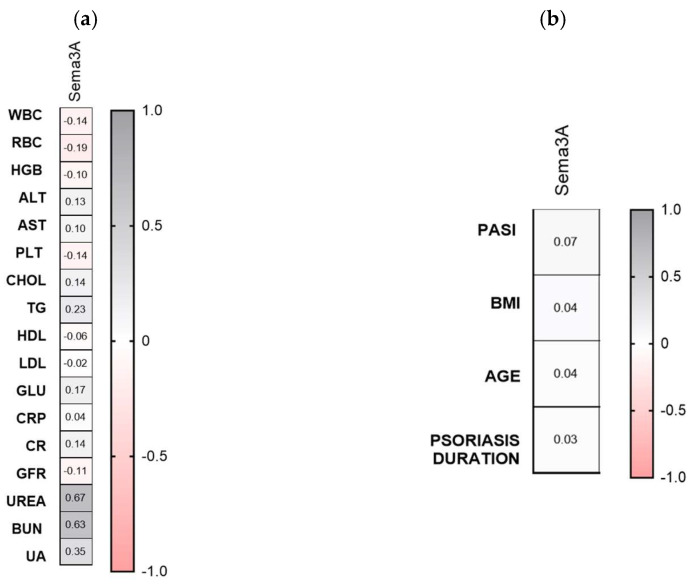
Correlations between serum semaphorin 3A concentrations and laboratory tests (**a**) or clinical parameters (**b**). TGs, triglycerides; ALT, alanine aminotransferase; AST, asparagine aminotransferase; GLU, fasting glucose; Chol, total cholesterol; HDL, high-density lipoprotein; LDL, low-density lipoprotein; RBC, red blood cells; WBC, white blood cells; PLT, platelets; HGB, hemoglobin; CR, creatinine. CON *n* = 30; PSOR *n* = 60.

With regard to links with laboratory parameters, significant positive correlations between Sema3A and UREA or BUN levels were demonstrated (R = 0.67, *p* = 0.01; R = 0.63, *p* = 0.02) (Figure 2a). Sema3A did not correlate with psoriasis severity, total BMI, psoriasis duration or age (NS) (Figure 2b).

### 3.2. Semaphorin 3E

**Figure 3:**
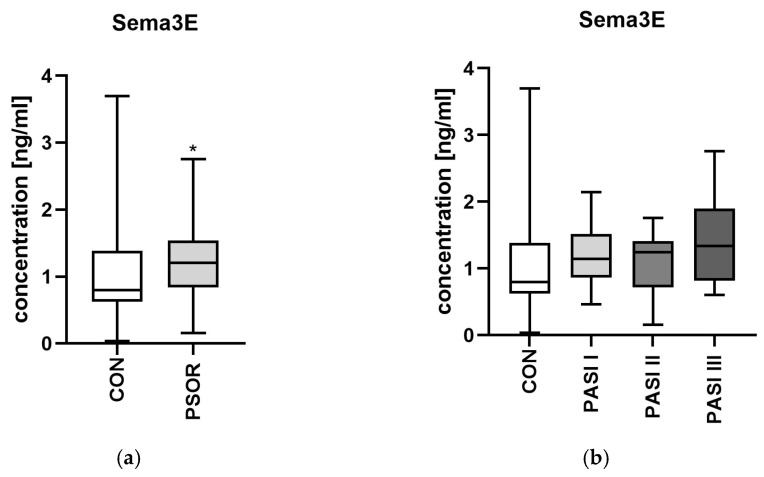

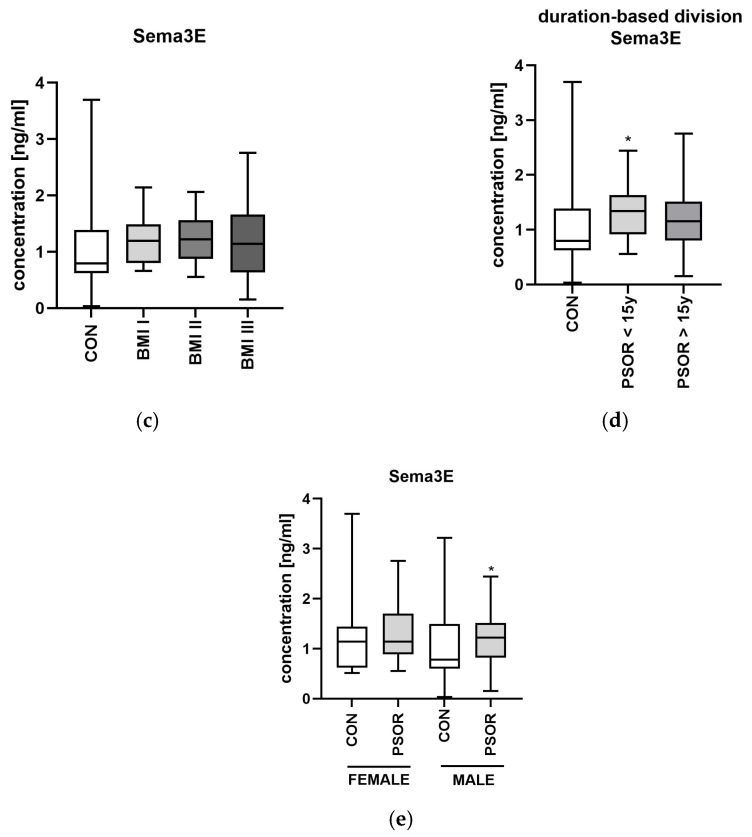
Serum semaphorin 3E concentration in patients in total (**a**) and divided regarding PASI (**b**), BMI (**c**), psoriasis duration (**d**), and gender (**e**) compared to controls. * means a statistically significant difference compared to the control group with *p* < 0.05 CON *n* = 30; PSOR *n* = 60.

Semaphorin 3E serum concentration was significantly higher in study group than in controls (*p* < 0.5) (Figure 3a).

No meaningful relations were found in terms of the PASI or BMI subdivision (Figure 3b,c). Patients with shorter-lasting disease and males had higher levels of SEMA3E than controls (*p* < 0.05) (Figure 3d,e).

**Figure 4:**
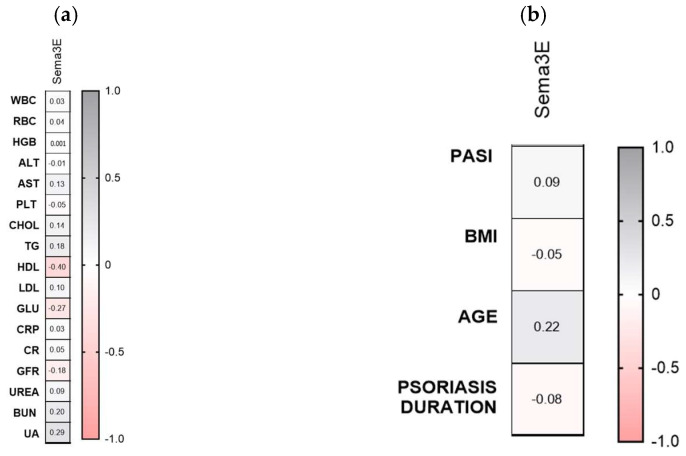
Correlations between serum Sema3E concentrations and laboratory tests (**a**) or clinical parameters (**b**). TGs, triglycerides; ALT, alanine aminotransferase; AST, asparagine aminotransferase; GLU, fasting glucose; Chol, total cholesterol; HDL, high-density lipoprotein; LDL, low-density lipoprotein; RBC, red blood cells; WBC, white blood cells; PLT, platelets; HGB, hemoglobin; CR, creatinine. CON *n* = 30; PSOR *n* = 60.

Sema3E significantly negatively correlated with HDL and glucose levels (R = −0.40, *p* = 0.01; R = −0.27, *p* = 0.04) (Figure 4a). Semaphorin 3E did not correlate with psoriasis severity, total BMI nor psoriasis duration or age (NS) (Figure 2b).

### 3.3. Semaphorin 4A

The serum concentration of semaphorin 4A was significantly strongly lower in psoriatic patienyts than controls (*p* < 0.001) (Figure 3a).

**Figure 5:**
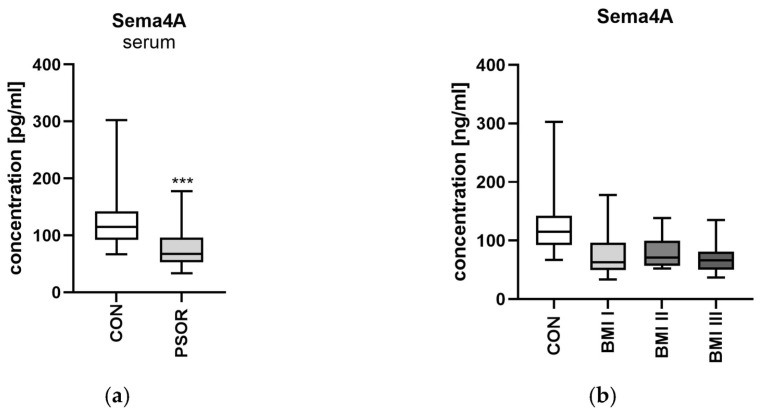

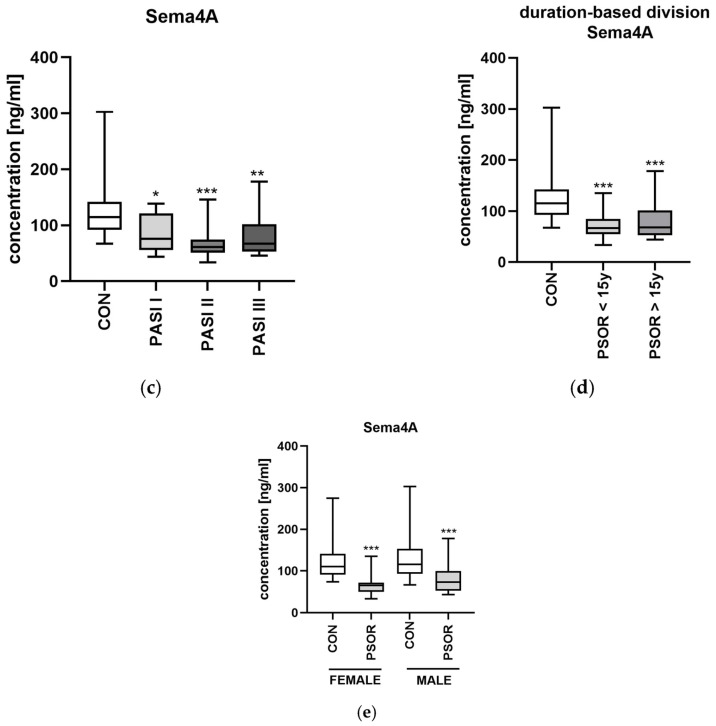
Serum semaphorin 4A concentration in patients in total (**a**) and divided regarding PASI (**b**), BMI (**c**), psoriasis duration (**d**), and gender (**e**) compared to controls. * means a statistically significant difference compared to the control group with *p* < 0.05 CON, ** *p* < 0.01 vs. CON, *** *p* < 0.001 vs. CON *n* = 30; PSOR *n* = 60.

**Figure 6:**
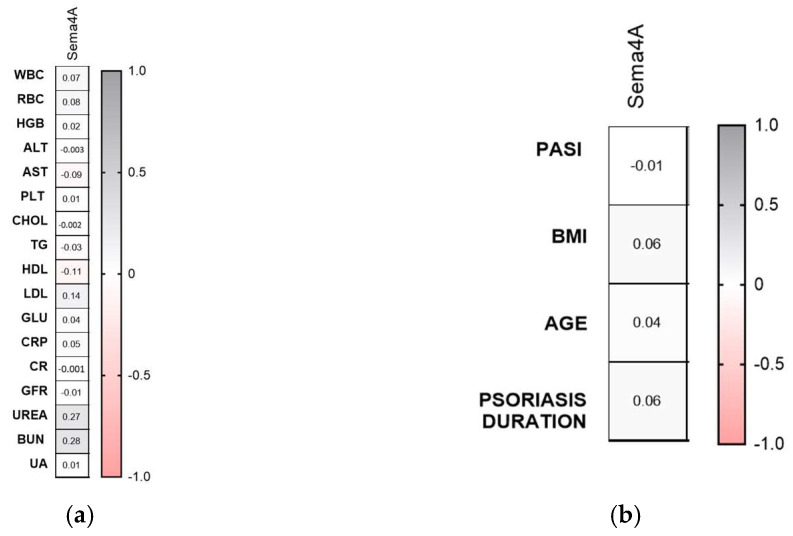
Correlations between serum semaphorin 4A concentrations and clinical parameters (**a**) or laboratory tests (**b**). TGs, triglycerides; ALT, alanine aminotransferase; AST, asparagine aminotransferase; GLU, fasting glucose; Chol, total cholesterol; HDL, high-density lipoprotein; LDL, low-density lipoprotein; RBC, red blood cells; WBC, white blood cells; PLT, platelets; HGB, hemoglobin; CR, creatinine.

The protein level did not differ regarding the BMI subgroups (Figure 5b). However, it did in the PASI subdivision: it was significantly lower in moderately and severe psoriatic patients (*p* < 0.0001, *p* < 0.01) (Figure 5c). Sema4A was significantly lower in controls regardless of the cut-off point in terms of disease duration or sex (Figure 5d,e). No specific relations between protein and the clinical or laboratory indices were noted (NS) (Figure 6a,b).

### 3.4. Semaphorin 4D

**Figure 7:**
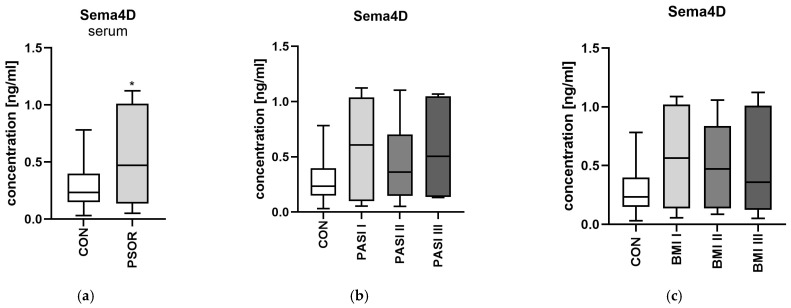
Serum semaphorin 4D concentration in patients in total (**a**) and divided regarding PASI (**b**) and BMI (**c**) compared to controls. * means a statistically significant difference compared to the control group with *p* < 0.05.

The serum concentration of semaphorin 4D was significantly higher in the study group than in the controls (*p* < 0.05) (Figure 7a). However, there were no relations between the protein and total PASI, BMI, age, psoriasis duration or within the PASI/BMI subgroups (NS) (Figure 7b,c). CON *n* = 30; PSOR *n* = 60.

**Figure 8:**
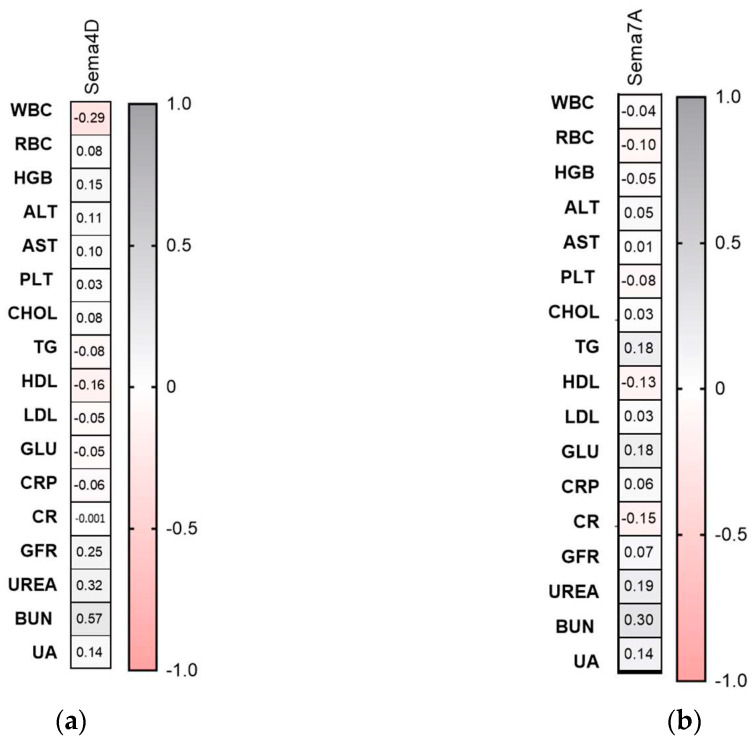
Correlations between serum semaphorin 4D (**a**) and semaphorin 7A (**b**) concentrations and laboratory tests. TGs, triglycerides; ALT, alanine aminotransferase; AST, asparagine aminotransferase; GLU, fasting glucose; Chol, total cholesterol; HDL, high-density lipoprotein; LDL, low-density lipoprotein; RBC, red blood cells; WBC, white blood cells; PLT, platelets; HGB, hemoglobin; CR, creatinine, UA, uric acid. CON *n* = 30; PSOR *n* = 60.

When analyzing links with laboratory parameters, Sema4D correlated significantly negatively with WBC and positively with BUN and UA (R = −0.29, *p* = 0.037; R = 0.57, *p* = 0.045; R = 0.14, *p* = 0.036) (Figure 8).

### 3.5. Semaphorin 7A

**Figure 9:**
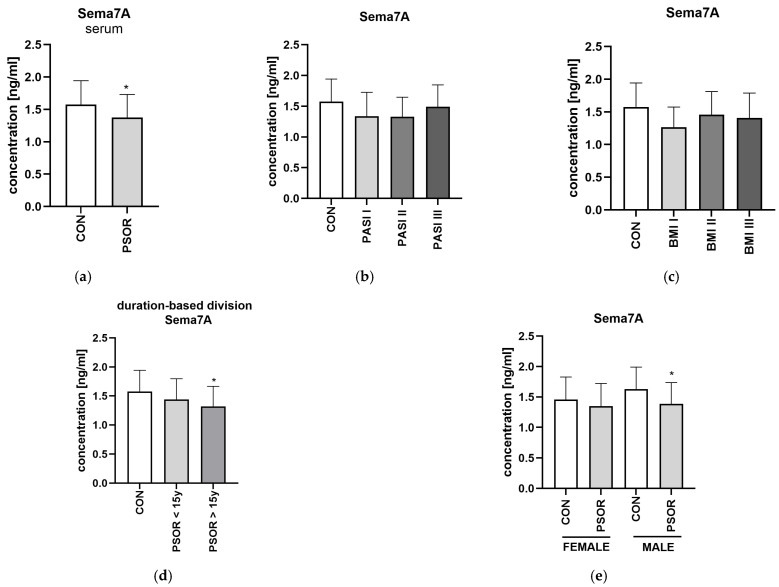
Serum semaphorin 7A concentration in patients in total (**a**) and divided regarding PASI (**b**), BMI (**c**), psoriasis duration (**d**), and gender (**e**) compared to controls. * means a statistically significant difference compared to the control group with *p* < 0.05.

Serum concentration of semaphorin 7A was significantly lower in patients with psoriasis than controls (*p* < 0.05) (Figure 9a). CON *n* = 30; PSOR *n* = 60.

Sema7A was not significantly related to total PASI, BMI, age, or disease duration (NS). Similarly, no important differences between the PASI or BMI subgroups were noted (Figure 9b,c). The semaphorin 7A level was significantly lower in patients with a longer history of psoriasis and male ones than in controls (Figure 9d,e). With regard to links with laboratory parameters, significant positive correlations between Sema7A and UREA or BUN levels were demonstrated (R = 0.67, *p* = 0.01; R = 0.63, *p* = 0.02). The protein did not correlate with psoriasis severity, total BMI, psoriasis duration, age or laboratory indices (NS).

## 4. Discussion

Numerous studies reported increased angiogenesis among patients with psoriasis, a process mediated by proinflammatory molecules including IL-8, IL-17 and TNF and mediators such as VEGF [17,18], which are observed in higher concentrations among psoriatic patients [17,18]. Some studies suggest that Sema3A might compete with VEGF and therefore exhibit antiangiogenic properties [19,20]. Kou et al. reported lower concentration of Sema3A in the skin samples taken from patients with psoriasis than in those taken from healthy volunteers [1]. Furthermore, they suggested another connection between this semaphorin and the psoriasis–itch–scratch cycle, a self-perpetuating mechanism where pruritus leads to mechanical damage that further increases the itch, which might be activated by lower Sema3A levels, but further studies are needed to elucidate the potential correlation [20]. In contrast, we observed a higher Sema3A concentration in serum among patients with psoriasis, which might reflect its role or its compensating effect in overexpressed angiogenesis in psoriasis. Regarding other autoimmune conditions, lower serum concentration of Sema3A was found among patients with systemic lupus erythematosus (SLE) than in the general population [21]. Moreover, Vadasz et al. noted a significant negative correlation between Sema3A level and the disease’s activity, which stays in line with finding from other papers that suggest the protein’s role in reducing autoimmune processes by downregulating T cell activity [21,22]. On the contrary, studies in malignant processes suggest that Sema3A decreases the migration of tumor-specific cytotoxic T tells into the tumor, which leads to its uncontrollable growth and invasion [23]. Sema3A seems to play an important role in other diseases: its elevated levels were linked to the development of atherosclerosis, observed in cerebral small vessel disease and linked to cognitive impairment [24,25]. In an animal model, Sema3A was also upregulated in obesity and DM, still common psoriatic comorbidities [26].

In our study, Sema3E was significantly higher among patients with psoriasis compared to the healthy volunteers. To our knowledge, this is the first study that evaluated this semaphorin in psoriasis. Increased levels of Sema3E, which were first identified in the nervous system as an axon guidance molecule, have been noted in other pathologic conditions, including idiopathic pulmonary fibrosis, cardiac diseases including sinus bradycardia and malignant processes such as colon cancer, metastatic breast cancer and melanoma [10,27]. On the contrary, decreased Sema3E concentration was observed in allergic asthma, which can be attributed to the fact that this molecule slows down the remodeling of the airways by decreasing the migration of smooth muscle cells [10]. Furthermore, some studies link the loss of function of the Sema3E mutation with cognitive disabilities, which further highlights its complex role [28]. We observed that Sema3E significantly negatively correlated with HDL and glucose levels. This might reflect its role in metabolic interplay in inflammatory-driven psoriasis. In some studies on animal models, the molecule was linked to the development of obesity and diabetes, which highlights the need for further exploration of the role of Sema3E in these diseases among psoriatic patients as well [29].

Sema4A is considered to be both pathogenic and therapeutic in allergies and malignant and autoimmune conditions due to its role in T helper differentiation, ability to increase CD8 T cell activity and amplify IL-17, which is a target side of many biological drugs used in psoriasis, including bimekizumab [8,,30]. We noted a significantly lower Sema4A concentration in patients with psoriasis compared to healthy individuals, and it was significantly lower in moderately and severe psoriatic patients, which suggests its protective role in this disease. No studies similar to ours can be found; however, Kume et al. noted lower Sema4A concentrations in keratinocytes in patients with psoriasis in both the psoriatic lesions and the non-lesional skin in comparison with the samples taken from individuals without this disease [8]. Moreover, they analyzed an animal model of psoriasis, where they observed more lesions in Sema4A knockout mice compared to wild mice [8]. The authors therefore concluded that Sema4A might play a role in psoriasis pathogenesis; however, since this molecule was only assessed in keratinocytes and no other cells, further studies are needed to get a comprehensive view on the involvement of Sema4A in this disease [8]. Furthermore, Sema4A seems to be able to modulate the spread of tumor cells in cancer development and their ability to metastasize, as well as reduce inflammation in allergic asthma in an animal model [31,32,33].

Sema4D plays a role in chemokine modulation and cell migration [33]. We found that Sema4D was significantly higher among patients with psoriasis compared to the control group. However, there were no relations between the protein and total PASI, BMI, age, psoriasis duration or within the PASI/BMI subgroups. No similar studies on this molecule can be found in the literature. Regarding other skin diseases, a positive correlation between Sema4D levels and the activity of oral lichen planus (OLP) was noted [33]. Ke et al. suggested that those correlations can be a result of Sema4D ability to modulate the activity of CD8+ T cells [33]. Moreover, the semaphorin seems to exhibit immunomodulatory functions not only by modulating T cell activity, but also cell adhesion and antibody production [34]. Increased Sema4D concentration was also noted in other diseases, including rheumatoid arthritis, liver fibrosis, retinopathy, and osteosarcoma, and is believed to be a negative predictor in myocardial infarction [35,36,37,38,39]. Furthermore, some studies suggest that Sema4D antagonists were found to be helpful in managing neurogenerative diseases including multiple sclerosis, Parkinsons’s and Alzheimer’s [40]. In an animal model, reduced Sema4D levels seem to increase glucose tolerance, but further studies are needed to assess its role among patients and in psoriatics [41].

Semaphorin 7A is believed to increase axon growth and activate monocytes and the production of IL-8 [5]. The concentration of semaphorin 7A was significantly lower among patients with psoriasis compared to the control group. Contrary to our findings, data suggest that Sema7A leads to monocyte activation and increased production of proinflammatory interleukins, which is typical for pathologic processes like psoriasis [5,16]. Overexpression of Sema7A was noted in diseases ranging from squamous cell carcinoma to acute aortic dissection [42,43]. However, some studies suggest that Sema7A can protect from hepatic steatosis by controlling lipogenesis and adipogenesis [44]. The absence of comparable studies constrains the depth of analysis yet simultaneously highlights emerging avenues that merit further investigation.

Our study has certain limitations, such as a study group that was not diverse enough, with a high male predominance and originating from one city and of one ethnicity. The limited number of individuals enrolled in the study could have led to a less representative group; therefore, we believe it would be valuable to consider further studies on a larger number of participants.

## 5. Conclusions

This study points to an association between semaphorins and psoriasis, which has not yet been comprehensively investigated. Individuals with psoriasis displayed a dysregulation of circulating semaphorins, characterized by elevated serum levels of Sema3A, Sema3E and Sema4D, accompanied by decreased concentrations of Sema4A and Sema7A relative to healthy controls. Sema3E, which significantly negatively correlated with HDL and glucose levels, could be potentially used to evaluate metabolic complications in psoriasis. In particular, Sema4A emerges as a candidate for further examination due to its potential involvement in the itch–scratch cycle, which represents a persistent clinical challenge for individuals with psoriasis and may ultimately inform the development of innovative therapeutic strategies. The semaphorins seem to be involved in psoriasis pathogenesis, but they are not related to its severity or clinical features such as BMI or age. Collectively, these results suggest that semaphorin alterations may mirror systemic perturbations associated with psoriasis and warrant further research.

## Data Availability

The original contributions presented in this study are included in the article. Further inquiries can be directed to the corresponding author.
